# Craniosynostosis in Patients With X‐Linked Hypophosphatemia: A Review

**DOI:** 10.1002/jbm4.10728

**Published:** 2023-03-14

**Authors:** Craig F Munns, Edward P Maguire, Angela Williams, Sue Wood, Andrew Biggin

**Affiliations:** ^1^ Mayne Academy of Paediatrics University of Queensland Brisbane Australia; ^2^ Child Health Research Centre University of Queensland Brisbane Australia; ^3^ Department of Endocrinology and Diabetes Queensland Children's Hospital Brisbane Australia; ^4^ 90TEN London UK; ^5^ Kyowa Kirin International Marlow UK; ^6^ Discipline of Child and Adolescent Health University of Sydney Sydney Australia; ^7^ Institute of Endocrinology and Diabetes The Children's Hospital at Westmead Westmead Australia

**Keywords:** CRANIOSYNOSTOSIS, FIBROBLAST GROWTH FACTOR 23, PHOSPHATE, SCAPHOCEPHALY, X‐LINKED HYPOPHOSPHATEMIA

## Abstract

Craniosynostosis is a rare condition of skull development, manifesting during fetal and early infant development, and is usually congenital. Craniosynostosis secondary to metabolic disorders, such as X‐linked hypophosphatemia (XLH), is less common and is typically diagnosed later than congenital craniosynostosis. XLH is a rare, progressive, and lifelong hereditary phosphate‐wasting disorder characterized by loss of function of the phosphate‐regulating endopeptidase homologue, X‐linked gene, which is associated with premature fusion of cranial sutures due to abnormal phosphate metabolism (hypophosphatemia) and altered bone mineralization or elevated levels of fibroblast growth factor 23. This targeted literature review of 38 articles seeks to provide an overview of craniosynostosis in individuals with XLH. The objectives of this review are to increase awareness of the prevalence, presentation, and diagnosis of craniosynostosis in XLH; examine the spectrum of craniosynostosis severity in XLH; discuss the management of craniosynostosis in those with XLH; recognize the complications for patients with XLH; and identify what is known about the burden of craniosynostosis for individuals with XLH. The presentation of craniosynostosis in individuals with XLH tends to manifest slightly later than congenital craniosynostosis and can vary in severity and appearance, making diagnosis difficult and resulting in inconsistent clinical outcomes. Consequently, craniosynostosis in patients with XLH is an underreported and potentially underrecognized condition. There have been no studies investigating the effects of craniosynostosis on the quality of life of people with XLH. Despite a growing awareness among researchers and experienced clinicians, there are still improvements to be made in general awareness and timely diagnosis of craniosynostosis in XLH. The XLH community would benefit from further study into the prevalence of craniosynostosis, the effect of XLH medical therapy on the development of craniosynostosis, and the effects of craniosynostosis on quality of life. © 2023 The Authors. *JBMR Plus* published by Wiley Periodicals LLC on behalf of American Society for Bone and Mineral Research.

## Graphical abstract

XLH is associated with an increased risk of craniosynostosis. Common presenting features are sagittal suture fusion, scaphocephaly, and altered head size/circumference. Frequent complications are elevated intracranial pressure, headaches, Chiari malformation type 1, papilledema, and syringomyelia. Diagnosis in XLH patients can be difficult; therefore, it is important to screen XLH patients for this disorder in the first 5 years of life.

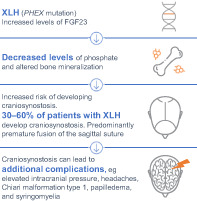

## Introduction

### Craniosynostosis

Craniosynostosis is a rare condition of skull development, with a reported prevalence of one in 1400 to 2100 children.^(^ ^1^ ^)^ Craniosynostosis is caused by premature fusion of one or more cranial sutures and can develop in utero or during infancy.^(^ ^2^ ^)^ In craniosynostosis, premature fusion of one or more cranial sutures means that, as the brain develops, the skull is unable to expand perpendicularly to the open sutures and instead has to expand in parallel to the fused sutures, resulting in an abnormal head shape.^(^ ^2^ ^)^ Primary or congenital craniosynostosis is typically diagnosed in infancy. It may be isolated and sporadic, or it may be associated with a genetic syndrome, such as Apert or Crouzon syndromes.^(^ ^1^ ^)^ Three‐quarters of genetically diagnosed cases are the result of mutations in one of six genes: fibroblast growth factor receptor 2 (*FGFR2*), fibroblast growth factor receptor 3 (*FGFR3*), twist family BHLH transcription factor 1 (*TWIST1*), ephrin B1 (*EFNB1*), transcription factor 12 (*TCF12*), and ETS2 repressor factor (*ERF*).^(^ ^1^ ^)^ Craniosynostosis secondary to metabolic disorders is less common and is typically diagnosed later than congenital craniosynostosis.^(^ ^3^ ^)^ The most common cause of metabolic craniosynostosis is hypophosphatemic rickets (HR), of which X‐linked hypophosphatemia (XLH) is the most common.^(^ ^4^, ^5^ ^)^ Craniosynostosis can also be present in nutritional rickets, possibly due to low serum phosphate.^(^ ^6^ ^)^

### XLH

XLH is a rare, hereditary, lifelong, deforming bone disorder with a prevalence of approximately one in 20,000 to 60,000.^(^ ^7^, ^8^, ^9^, ^10^ ^)^ The disorder is caused by inactivating mutations in the phosphate‐regulating endopeptidase homologue, X‐linked (*PHEX*) gene.^(^ ^2^, ^11^ ^)^ XLH is characterized by excess levels of circulating fibroblast growth factor 23 (FGF23), resulting in increased renal phosphate excretion, reduced intestinal phosphate absorption, and chronic hypophosphatemia.^(^ ^2^ ^)^ This in turn leads to defective bone and tooth mineralization and formation, causing rickets, osteomalacia, and odontomalacia in children, in addition to several other skeletal and extraskeletal symptoms.^(^ ^2^, ^11^ ^)^

The earliest report associating craniosynostosis with likely XLH (the case of HR was described as vitamin D‐resistant rickets) was in 1951 by Olga Imerslund.^(^ ^12^ ^)^ Further cases of craniosynostosis were reported in 1954 by Coleman and Foote and in 1958 by Hugh‐Jones and Harris,^(^ ^13^, ^14^ ^)^ and there were then no further reports in the literature until the 1980s, when more case studies were published; the first reference to craniosynostosis and XLH in the 1980s was made by Carlsen and colleagues.^(^ ^15^ ^)^ A development in the understanding of craniosynostosis came from the Hyp mutant mouse, a murine model of XLH that displays similar characteristics to XLH in humans, including the development of craniosynostosis.^(^ ^16^, ^17^ ^)^ From the 1980s onward, the awareness of craniosynostosis associated with XLH grew, and there is now a greater understanding of the prevalence, characteristics, and complications of craniosynostosis in patients with XLH (Table 1).

**Table 1:** Search Results

Title	Primary author(s)	Article type	Citation	Publication year
Craniostenosis and vitamin D resistant rickets	Imerslund O	Case study	Acta Paediatr. 1951;40(5):449–56.^(^ ^12^ ^)^	1951
Craniostenosis with familial vitamin‐D‐resistant rickets	Coleman EN, Foote JB	Case study	Br Med J. 1954;1(4861):561–2.^(^ ^13^ ^)^	1954
Vitamin‐D‐resistant rickets with craniostenosis	Hugh‐Jones K, Harris CF	Case study	Proc R Soc Med. 1958;51(9):740–1.^(^ ^14^ ^)^	1958
Craniometric measurements of craniofacial malformations in mice with X‐linked, dominant hypophosphatemia (vitamin D‐resistant rickets)	Iorio RJ	Original empirical study	Teratology. 1980;22(3):291–8.^(^ ^16^ ^)^	1980
Craniofacial synostosis in association with vitamin D–resistant rickets	McCarthy JG, Reid CA	Case study	Ann Plast Surg. 1980;4(2):149–53.^(^ ^37^ ^)^	1980
Craniosynostosis in vitamin D‐resistant rickets. A mouse model	Roy WA	Original empirical study	J Neurosurg. 1981;55(2):265–71.^(^ ^17^ ^)^	1981
Premature cranial synostosis in X‐linked hypophosphatemic rickets: possible precipitation by 1‐alpha‐OH‐cholecalciferol intoxication	Carlsen NLT	Case study	Acta Paediatr Scand. 1984;73(1):149–54.^(^ ^15^ ^)^	1984
Premature cranial synostosis and hypophosphatemic rickets	Clemens P	Letter to the editor/retrospective study	Acta Paediatr Scand. 1984;73(6):857.^(^ ^32^ ^)^	1984
Chiari I malformation: association with hypophosphatemic rickets and MR imaging appearance	Caldemeyer KS	Prospective study	Radiology. 1995;195(3):733–8.^(^ ^41^ ^)^	1995
Craniosynostosis in X‐linked hypophosphataemic rickets	Willis FR, Beattie TJ	Case series and review	J Paediatr Child Health. 1997;33(1):78–9.^(^ ^5^ ^)^	1997
Chiari malformation associated with vitamin D‐resistant rickets: case report	Kuether TA, Piatt JH	Case study	Neurosurgery. 1998;42(5):1168–71.^(^ ^42^ ^)^	1998
Early treatment improves growth and biochemical and radiographic outcome in X‐linked hypophosphatemic rickets	Mäkitie O	Retrospective study	J Clin Endocrinol Metab. 2003;88(8):3591–7.^(^ ^25^ ^)^	2003
The role of cranial expansion for craniocephalic disproportion	Gough J	Case series	Pediatr Neurosurg. 2005;41(2):61–9.^(^ ^22^ ^)^	2005
Sagittal synostosis in X‐linked hypophosphatemic rickets and related diseases	Currarino G	Retrospective study	Pediatr Radiol. 2007;37(8):805–12.^(^ ^18^ ^)^	2007
X‐linked hypophosphatemic rickets and craniosynostosis	Murthy AS	Case study	J Craniofac Surg. 2009;20(2):439–42.^(^ ^20^ ^)^	2009
Papilledema in the setting of X‐linked hypophosphatemic rickets with craniosynostosis	Glass LR	Case study	Case Rep Ophthalmol. 2011;2(3):376–81.^(^ ^24^ ^)^	2011
Age at initial consultation for craniosynostosis: comparison across different patient characteristics	Seruya M	Retrospective study	J Craniofac Surg. 2013;24(1):96–8.^(^ ^29^ ^)^	2013
Patulous subarachnoid space of the optic nerve associated with X‐linked hypophosphatemic rickets	Galvez‐Ruiz A, Chaudhry I	Case study/photo essay	Neuro‐Ophthalmology. 2013;37(3):129–32.	2013
Bilateral coronal and sagittal synostosis in X‐linked hypophosphatemic rickets: a case report	Freudlsperger C	Case study	J Craniomaxillofac Surg. 2013;41(8):842–4.^(^ ^36^ ^)^	2013
Therapeutic management of hypophosphatemic rickets from infancy to adulthood	Linglart A	Review	Endocr Connect. 2014;3(1):R13–30.	2014
Copper beaten skull in X‐linked hypophosphataemic rickets	Ram R	Images in nephrology article (brief clinical report)	Clin Exp Nephrol. 2015;19(1):157.^(^ ^46^ ^)^	2015
X‐linked hypophosphatemic rickets and sagittal craniosynostosis: three patients requiring operative cranial expansion: case series and literature review	Jaszczuk P	Case series and literature review	Childs Nerv Syst. 2016;32(5):887–91.^(^ ^4^ ^)^	2016
Hypophosphatemic rickets and craniosynostosis: a multicenter case series	Vega RA	20‐year retrospective study	J Neurosurg Pediatr. 2016;17(6):694–700.^(^ ^3^ ^)^	2016
Clinical genetics of craniosynostosis	Wilkie AOM	Review	Curr Opin Pediatr. 2017;29(6):622–8.^(^ ^1^ ^)^	2017
Craniosynostosis as the presenting feature of X‐linked hypophosphatemic rickets	Vakharia JD	Case series	Pediatrics. 2018;141(Suppl 5):S515–S9.^(^ ^23^ ^)^	2018
High incidence of cranial synostosis and Chiari I malformation in children with X‐linked hypophosphatemic rickets (XLHR)	Rothenbuhler A	Retrospective study	J Bone Miner Res. 2019;34(3):490–6.^(^ ^33^ ^)^	2019
The first Korean case report with scaphocephaly as the initial sign of X‐linked hypophosphatemic rickets	Lee KS, Lee BL	Case study	Childs Nerv Syst. 2019;35(6):1045–9.^(^ ^21^ ^)^	2019
Clinical practice recommendations for the diagnosis and management of X‐linked hypophosphataemia	Haffner D	Panel and Delphi method–based review to form clinical practice recommendations (grading matrix and PICO [patient/problem, intervention, comparison, outcome] were utilized)	Nat Rev Nephrol. 2019;15(7):435–55.^(^ ^2^ ^)^	2019
Craniosynostosis and metabolic bone disorder. A review	Di Rocco F	Review	Neurochirurgie. 2019;65(5):258–63.^(^ ^19^ ^)^	2019
X‐linked hypophosphatemia: management and treatment prospects	Lambert AS	Review	Joint Bone Spine. 2019;86(6):731–8.	2019
Phenotypes of a family with XLH with a novel *PHEX* mutation	Yamamoto A	Case study	Hum Genome Var. 2020;7:8.^(^ ^39^ ^)^	2020
Twin girls with hypophosphataemic rickets and papilloedema	Migliarino V	Case study with clinical education questions and answers	Arch Dis Child Educ Pract Ed. 2022;107(2):124–6.^(^ ^38^ ^)^	2020
Mineralized tissues in hypophosphatemic rickets	Robinson ME	Review	Pediatr Nephrol. 2020;35(10):1843–54.	2020
Hereditary hypophosphatemic rickets and craniosynostosis	Arenas MA	Descriptive and retrospective study	J Pediatr Endocrinol Metab. 2021;34(9):1105–13.^(^ ^31^ ^)^	2021
X‐linked hypophosphatemic rickets: multisystemic disorder in children requiring multidisciplinary management	Baroncelli GI, Mora S	Review article	Front Endocrinol (Lausanne). 2021;12:688309.	2021
Magnetic resonance imaging is a valuable tool to evaluate the therapeutic efficacy of burosumab in children with X‐linked hypophosphatemia	Zhukouskaya VV	Prospective longitudinal open cohort study	Eur J Endocrinol. 2021;185(4):475–84.^(^ ^35^ ^)^	2021
X‐linked hypophosphatemia and burosumab: practical clinical points from the French experience	Bacchetta J	Panel and consensus–based review to form guidance for diagnosis, treatment, and management of pediatric patients with XLH on burosumab treatment	Joint Bone Spine. 2021;88(5):105208.	2021
Monitoring response to conventional treatment in children with XLH: value of ALP and Rickets Severity Score (RSS) in a real world setting	Uday S	Longitudinal multicenter retrospective study	Bone. 2021;151:116025.	2021

Craniosynostosis has also been noted to be present in other hypophosphatemic diseases caused by excess levels of FGF23: autosomal dominant hypophosphatemic rickets^(^ ^3^, ^18^ ^)^ and a possible case of cutaneous‐skeletal hypophosphatemia syndrome.^(^ ^18^ ^)^

### Pathogenesis of craniosynostosis in XLH

It has been theorized that the loss of function of *PHEX* in XLH leads to premature fusion of the cranial sutures due to abnormal phosphate metabolism (hypophosphatemia) and altered bone mineralization and/or the elevated levels of FGF23 found in individuals with XLH.^(^ ^4^, ^17^, ^19^ ^)^

The levels of serum phosphate in a newborn with XLH are typically normal and do not start to decrease until 3 to 12 months of age.^(^ ^2^, ^20^ ^)^ This is consistent with the later onset of craniosynostosis observed in patients with XLH compared with those with congenital craniosynostosis.

FGF23 can bind to both FGFR2 and FGFR3, and it has been suggested that FGF23 can cross‐bind FGFR2 and FGFR3 at the cranial sutures, causing premature fusion.^(^ ^20^ ^)^ This is consistent with the role of mutations in *FGFR2* and *FGFR3* in congenital craniosynostosis.

However, exactly how XLH leads to craniosynostosis has not yet been determined. For example, it has been observed that specific *PHEX* mutations have limited predictive value for the development of craniosynostosis,^(^ ^21^ ^)^ and there are rare instances of craniosynostosis occurring postnatally in patients with XLH^(^ ^4^, ^21^, ^22^, ^23^ ^)^ before the development of hypophosphatemia. Furthermore, although treating XLH with the conventional therapy of active vitamin D analogues (eg, calcitriol, alphacalcidol) and phosphate supplements may improve serum phosphate levels enough to ameliorate rickets in children, this does not appear to be able to prevent craniosynostosis,^(^ ^20^, ^24^, ^25^ ^)^ and there is a lack of evidence on the effect of burosumab, a fully humanized immunoglobulin G antibody to FGF23, on the development of craniosynostosis. Robust studies are needed to assess whether pharmacological treatments can reduce the severity or prevalence of craniosynostosis in these patients. Such studies could provide information on the respective roles that FGF23 and hypophosphatemia play in craniosynostosis development in children with XLH. The effect of burosumab on osteoblasts and osteoclasts is being assessed in two clinical trials (HYPO‐BLASTES and HYPO‐CLASTE).^(^ ^26^, ^27^ ^)^ Bone cells from patients with HR who are also receiving burosumab and/or 1,25(OH)_2_D and controls will be harvested via blood samples in HYPO‐CLASTE and from patients undergoing corrective surgery for craniosynostosis in HYPO‐BLASTES. In the HYPO‐BLASTES study, cultured osteoblasts from patients with HR and craniosynostosis will be compared with individuals with idiopathic craniosynostosis (estimated primary completion quarter two 2024).^(^ ^26^, ^27^, ^28^ ^)^

Unless there is routine screening, recognition of craniosynostosis may be dependent on parents and pediatricians visually recognizing craniofacial deformity.^(^ ^29^ ^)^ However, there is a large variability in the appearance of craniosynostosis, which can make initial diagnosis difficult.^(^ ^29^ ^)^ Craniosynostosis is important to diagnose early in its development because, if left untreated, complications such as elevated intracranial pressure (ICP) with neurological sequelae may develop.^(^ ^29^ ^)^ Treatment is dependent on the severity of cranial deformity, the complications, and the patient's age, and includes cranial surgery and cranial helmets.^(^ ^22^, ^30^ ^)^

The current review of craniosynostosis in patients with XLH has been carried out to examine the level of awareness in the field and provide an overview of craniosynostosis in patients with XLH. The objectives are to increase awareness among clinicians of the prevalence, presentation, diagnosis, and complications of craniosynostosis in XLH; examine the spectrum of craniosynostosis severity in XLH; review the management of craniosynostosis in XLH; and identify what is known about the burden of craniosynostosis for XLH patients. This review will also highlight areas for future research.

## Materials and Methods

A targeted literature review was conducted of the PubMed database from September 24–30, 2021. Records were identified using the following search terms: (XLH) OR (X‐linked hypophosphatemia) OR (X‐linked dominant hypophosphatemic rickets) OR (X‐linked hypophosphatemic rickets) OR (familial hypophosphatemia) OR (heritable hypophosphatemic rickets) OR (X‐linked vitamin D‐resistant rickets) OR (vitamin D‐resistant rickets) AND (craniosynostosis) OR (scaphocephaly) OR (dolichocephaly) OR (anterior plagiocephaly) OR (posterior plagiocephaly) OR (trigonocephaly) OR (brachycephaly) OR (oxycephaly) OR (Apert) OR (Crouzon) OR (Pfeiffer) OR (Muenke) OR (Kleeblattschade) OR (premature fusion) OR (frontal bossing) OR (cranial suture) OR (craniostenosis). Records were stored using a citation manager (Endnote X9.3.3, Clarivate, Philadelphia, PA, USA). We screened all titles and abstracts, and, after assessment, full texts were examined to evaluate eligibility. Full articles were screened by a single reviewer for relevance. Eligibility criteria were simply the inclusion of craniosynostosis, or associated terms, in relation to patients with XLH, or related terms, within literature reviews, case studies, or studies, and English as the article language. In addition, we manually screened the bibliographies of eligible texts to identify potentially relevant literature.

## Results

**Fig. 1:**
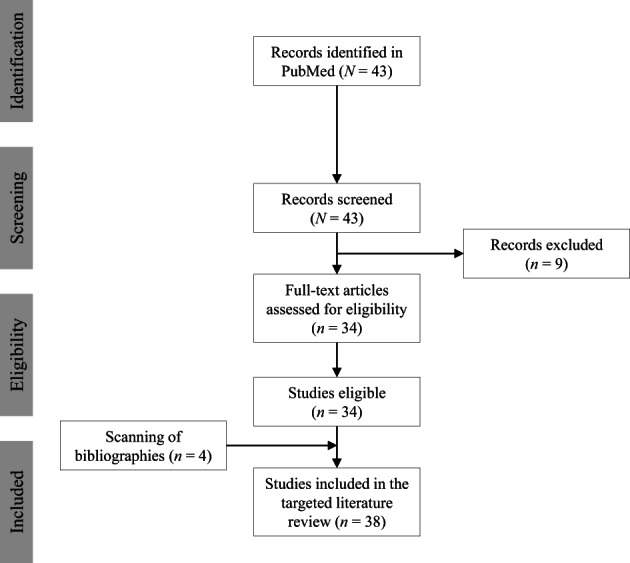
Study selection flow diagram.

The literature search yielded 43 records, the titles and abstracts of which were screened. Nine records were excluded because they were not relevant; 34 full‐text articles were eligible. Additional inspection of the references of these articles identified four more relevant articles. Overall, 38 articles were identified that cited craniosynostosis, or related terms, in patients with XLH (Fig. 1). These included 20 case studies, eight small‐center studies, six review articles, and six papers that mentioned craniosynostosis, with some articles covering more than one of these elements.

When the search terms for craniosynostosis were excluded, the literature search yielded 3080 records. Only 1.4% of these records were available when craniosynostosis search terms were included, suggesting that the awareness of craniosynostosis in patients with XLH is low in the field.

In total, 92 patients were identified to have XLH and craniosynostosis. This is likely to be an underestimation; some publications did not differentiate between XLH and hereditary hypophosphatemia, and only patients with confirmed XLH were counted in this review of the literature.

### Epidemiology

The literature search identified only five publications that reported on the number of patients with XLH who had craniosynostosis.^(^ ^18^, ^25^, ^31^, ^32^, ^33^ ^)^ Clemens described the presence of craniosynostosis in 2 of the 6 patients with XLH, but this was a small sample size from one clinic.^(^ ^32^ ^)^ Mäkitie and colleagues reported that 2 of 19 patients with XLH underwent surgery for craniosynostosis, which is a reflection of the severity of craniosynostosis within an XLH population rather than providing an understanding of the prevalence of craniosynostosis.^(^ ^25^ ^)^ Currarino reported that 13 of 28 patients with HR also had craniosynostosis, but this study included patients other than those with a diagnosis of XLH.^(^ ^18^ ^)^ Rothenbuhler and colleagues undertook a systematic investigation of craniosynostosis in an XLH cohort from a single center in France. The investigators accessed the computerized tomography scans of their entire patient cohort (*n* = 44) to determine the presence of craniosynostosis. Genetic analysis confirmed a *PHEX* mutation in most cases (*n* = 36; 82%). They were able to include asymptomatic craniosynostosis cases and reported that 59% (26 of 44 patients with XLH) had craniosynostosis. They also found that craniosynostosis was significantly associated with a history of dental abscesses.^(^ ^33^ ^)^ Arenas and colleagues reported the occurrence of craniosynostosis in a center in Argentina. In this study, the occurrence was determined by reviewing the skull imaging of patients with hereditary HR, and it was found that 52% of patients (26 of 50) had craniosynostosis, most of whom were asymptomatic. Although the phenotype of the cohort was consistent with XLH, genetic testing for a *PHEX* mutation was performed in only 14 patients, with a further 3 having a family history supportive of X‐linked inheritance.^(^ ^31^ ^)^ It was not stated if those with craniosynostosis had a confirmed *PHEX* mutation.^(^ ^31^ ^)^

XLH is a dominant X‐linked disease with a higher prevalence in the female population.^(^ ^34^, ^35^ ^)^ Notably, the reported occurrence of craniosynostosis is disproportionately more frequent in males than females, which is supported by the cases noted in this literature review (35 males; 25 females) (Table 2). Arenas and colleagues reported 15 cases of males and 11 of females with craniosynostosis. However, as there were 50% more females in the study than males, the percentage of males with craniosynostosis was 75% (15 of 20) compared with 37% (11 of 30) of females.^(^ ^31^ ^)^ The finding that there are more males than females with craniosynostosis is consistent with findings from an animal model of XLH, which found that male craniofacial malformation was more severe than that found in females.^(^ ^16^ ^)^ Although it is not known if craniosynostosis is also more severe in human males, Vega and colleagues report that males exhibited a more complex craniosynostosis with higher clinical compromise,^(^ ^3^ ^)^ suggesting that this may be the case. It is possible that the non‐mutated *PHEX* gene in heterozygous females exerts a protective effect.^(^ ^16^ ^)^

**Table 2:** Patients With XLH and Craniosynostosis

Primary author(s)	Year of publication	Patient sex	Age of craniosynostosis diagnosis	Fused sutures	Symptoms/presentation	Treatment	Occurrence
Imerslund O	1951	M	1 year	Sagittal	Scaphocephaly, papilledema, elevated ICP	Surgery	
Coleman EN, Foote JB	1954	M	20 months	Sagittal	Scaphocephaly	Unknown	
Hugh‐Jones K, Harris CF	1958	M	18 months	All apart from coronal	Frontal bossing, skull thickening	None	
McCarthy JG, Reid CA	1980	F	11 months	Pansynostosis	Frontal bossing at birth	Surgery	
Carlsen NLT	1984	M	14 months	Sagittal (?)	Scaphocephaly, elevated ICP	None	
Clemens P	1984	Unknown	2 years	Unknown	Unknown	Unknown	2/6 (33.3%)
		Unknown	2 years	Unknown	Unknown	Unknown	
Willis FR, Beattie TJ	1997	M	36 months	Pansynostosis	Papilledema, strabismus	Surgery	3/3 (100%)
		M	5 years	Sagittal	Scaphocephaly, elevated ICP	Surgery	
		M	42 months	Sagittal	Headaches, papilledema, strabismus	Surgery	
Mäkitie O	2003	Unknown	Unknown	Unknown	Unknown	Surgery	2/19 (10.5%)
		Unknown	Unknown	Unknown	Unknown	Surgery	
Gough J	2005	M	5 months	Metopic	Papilledema, elevated ICP, headaches, CM1, seizures	Surgery	
Currarino G	2007	F	8 years 11 months	Unknown	Unknown	Unknown	13/28 (46.4%)^a^
		F	7 years	Unknown	Unknown	Unknown	
		F	9 years 6 months	Unknown	Unknown	Unknown	
		M	5 years 6 months	Unknown	Unknown	Unknown	
		F	2 years 6 months	Unknown	Unknown	Unknown	
		M	1 year 6 months	Unknown	Unknown	Unknown	
		F	4 years	Unknown	Unknown	Unknown	
		M	5 years 6 months	Unknown	Unknown	Unknown	
		F	2 years 7 months	Unknown	Unknown	Unknown	
		M	4 years	Unknown	Unknown	Unknown	
		F	2 years 8 months	Unknown	Unknown	Unknown	
Murthy AS	2009	M	1 year 11 months	Sagittal	Papilledema, elevated ICP	Surgery	
Glass LR	2011	F	3 years	Sagittal and metopic fused, others nearly closed	Papilledema, elevated ICP, scaphocephaly	Surgery	
Freudlsperger C	2013	M	18 months	Turribrachycephalic skull	Turribrachycephalic skull	Surgery	
Jaszczuk P	2016	F	15 months	Sagittal	Scaphocephaly, papilledema, elevated ICP	Surgery	
		M	2 years	Sagittal, metopic	Scaphocephaly, papilledema, elevated ICP, frontal bossing	Surgery	
		F	3 years	Sagittal	Bony protrusion from anterior fontanelle, headaches, elevated ICP	Surgery	
Vega RA	2016	M	18 months	Sagittal	Scaphocephaly, headaches	Surgery	
		M	3 years	Pansynostosis	Brachycephaly, elevated ICP, headaches, CM1	Surgery	
		M	5 years	Sagittal	Worsened school performance, headaches	None	
		F	4 years	Sagittal	Frontal bossing, scaphocephaly, papilledema, elevated ICP, CM1	Surgery	
		M	9 years	Pansynostosis	CM1, syringomyelia, elevated ICP, unusual skull shape	Surgery	
		F	4 years	Sagittal	Scaphocephaly	Surgery	
		M	2 years	Sagittal	Scaphocephaly, papilledema	Surgery	
		M	13 months	Sagittal	Scaphocephaly	Surgery	
Vakharia JD	2018	F	3 months	Sagittal	Unknown	Surgery	
		M	3 months	Sagittal	Scaphocephaly, frontal bossing, sagittal ridging	None	
Rothenbuhler A	2019	26 (M & F)	Unknown	Unknown	Unknown^b^	Unknown	26/44 (59.1%)
Lee KS, Lee BL	2019	M	11 months	Unknown	Scaphocephaly	Surgery	
Yamamoto A	2020	M	4 years 11 months	Unknown	CM1	Unknown	
Migliarino V	2022	F	7 years	Sagittal	Strabismus, papilledema, high forehead	Surgery	
		F	7 years	Sagittal	Papilledema	Surgery	
Arenas MA	2021	F	3 years 7 months	Sagittal, lambdoid, right coronal	Cranial asymmetry, elevated ICP	Surgery	26/50 (52.0%)^a^
		F	Unknown	Sagittal	Scaphocephaly	None	
		F	Unknown	Sagittal, lambdoid	Scaphocephaly	None	
		F	Unknown	Sagittal	Scaphocephaly, CM1	None	
		F	Unknown	Pansynostosis	Scaphocephaly	None	
		F	Unknown	Sagittal	None	None	
		F	Unknown	Sagittal	Scaphocephaly	None	
		F	Unknown	Sagittal	Scaphocephaly	None	
		F	Unknown	Sagittal	Scaphocephaly	None	
		M	Unknown	Sagittal	Scaphocephaly	None	
		M	Unknown	Pansynostosis	None	None	
		M	Unknown	Sagittal	None	None	
		M	Unknown	Pansynostosis	Scaphocephaly	None	
		M	5 years 6 months	Pansynostosis	Macrocephaly, CM1, headaches, elevated ICP	Surgery	
		M	Unknown	Pansynostosis	None	None	
		M	6 months	Sagittal	None	Surgery	
		M	Unknown	Sagittal	Scaphocephaly	None	
Uday S	2021	M	Unknown	Unknown	Unknown	Unknown	
		M	Unknown	Unknown	Unknown	Unknown	
		M	Unknown	Unknown	Unknown	Unknown	

Abbreviation: CM1 = Chiari malformation type 1; F = female; HR = hypophosphatemic rickets; ICP = intracranial pressure; M = male; XLH = X‐linked hypophosphatemia.

^a^ Includes all patients with HR, not only patients with XLH.

^b^ One patient with craniosynostosis and CM1 experienced headaches.

### Presentation and diagnosis of craniosynostosis in XLH

Craniosynostosis in children with XLH tends to present later than in those with primary/congenital craniosynostosis^(^ ^29^ ^)^; this may be because hypophosphatemia has not been reported in neonates until 3 to 4 months of age^(^ ^2^ ^)^ or 6 to 12 months of age.^(^ ^20^ ^)^ Therefore, the prevalence of craniosynostosis in XLH may be underreported, as late/mild cases may go unnoticed. Seruya and colleagues found that the median age of presentation with craniosynostosis was 4.1 months for all patients. In contrast, the 3 patients with XLH from this study presented with craniosynostosis at a much later age: median 32.3 months.^(^ ^29^ ^)^

**Fig. 2:**
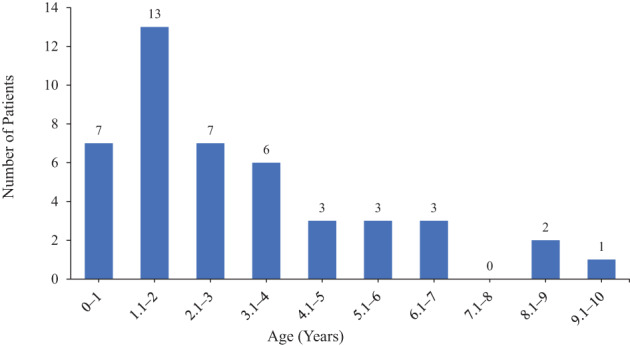
Age distribution of craniosynostosis diagnosis in patients with XLH identified in this review. Note, the age of craniosynostosis diagnosis was not reported for all patients; this information was only available for 45 of 92 patients. XLH = X‐linked hypophosphatemia.

The median age of diagnosis of craniosynostosis in patients with XLH in this literature review is similar to that reported for patients with XLH by Seruya and colleagues (*n* = 3).^(^ ^29^ ^)^ The age range in this review is from 3 months to 9 years and 6 months, and the median age of diagnosis is 2 years and 7.5 months (*n* = 42, Table 2 and Fig. 2).^(^ ^3^, ^4^, ^5^, ^12^, ^13^, ^14^, ^15^, ^18^, ^20^, ^21^, ^22^, ^23^, ^24^, ^31^, ^32^, ^36^, ^37^, ^38^, ^39^ ^)^ There are, however, reports of children with XLH born with features of craniosynostosis.^(^ ^21^, ^23^ ^)^

### Presenting features of craniosynostosis

**Fig. 3:**
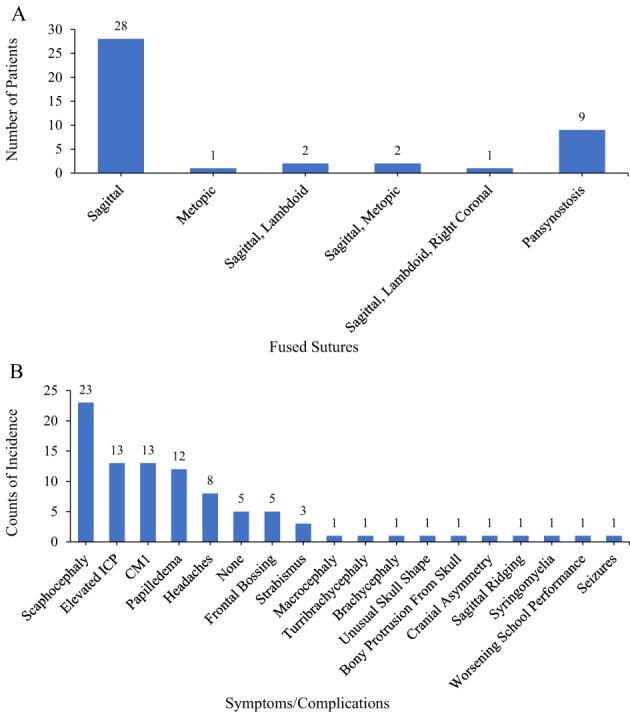
(*A*) Distribution of fused sutures and (*B*) distribution of symptoms/complications in craniosynostosis in patients with XLH identified in this review. Note, the sutures fused were not reported for all patients; this information was only available for 43 of 92 patients. The distribution of symptoms/complications was also not reported for all patients, only being available for 47 of 92 patients. Collection of these data cannot be assumed to be complete for the patients included because of limitations in diagnostic tests and awareness of symptoms/complications associated with craniosynostosis in patients with XLH at the time of diagnosis. CM1 = Chiari malformation type 1; ICP = intracranial pressure; XLH = X‐linked hypophosphatemia.

The most common feature of craniosynostosis in patients with XLH is fusion of the sagittal suture and scaphocephaly (Fig. 3 *A*, *B*).^(^ ^3^, ^4^, ^5^, ^12^, ^13^, ^15^, ^21^, ^23^, ^24^, ^31^ ^)^ However, if craniosynostosis develops at a later stage, there may not be scaphocephaly present, and the sagittal suture may fuse without any obvious signs. In such cases, the fused suture is only detectable radiologically,^(^ ^19^ ^)^ as is often the case in patients with XLH.^(^ ^33^ ^)^ Scaphocephaly is not the only cranial deformity that can occur in craniosynostosis in patients with XLH; for example, pansynostosis with brachycephaly has also been reported as a presenting feature.^(^ ^36^ ^)^

Altered head size is a common feature of craniosynostosis in patients with XLH, as the skull cannot expand normally with brain growth.^(^ ^2^ ^)^ Both increased and decreased circumferences have been reported, with increased head circumference being more common.^(^ ^4^, ^12^, ^21^, ^31^ ^)^ The abnormal head shape with frontal bossing that occurs in XLH is independent of craniosynostosis.^(^ ^2^, ^4^ ^)^ However, patients can present with a normal cranial circumference; this may depend on which sutures have fused, when the fusion occurred, and the age of the patient when examined.^(^ ^31^ ^)^

Elevated ICP can result in papilledema or strabismus.^(^ ^3^, ^38^ ^)^ Patients with raised ICP also report frequent headaches and vomiting or can be found to have bulging of the anterior fontanel.^(^ ^2^, ^4^ ^)^

### Diagnosis of craniosynostosis in XLH

The signs and symptoms of craniosynostosis should be sought at each clinical visit. Once features of craniosynostosis have been recognized, confirmation with computerized tomography imaging can be undertaken. This will also allow investigation for the presence of elevated ICP and Chiari malformation type 1 (CM1). Magnetic resonance imaging (MRI) scans provide more detailed brain and spine imaging for evaluation of possible CM1 and syringomyelia; fundoscopy, functional MRI, or optical coherence tomography can be used to screen for papilloedema.^(^ ^19^, ^40^ ^)^ Difficulties arise when craniosynostosis is later developing, more subtle in appearance, or even asymptomatic or normocephalic because there may be no external signs of craniosynostosis.^(^ ^3^, ^31^, ^38^ ^)^ Consequently, the true prevalence of craniosynostosis may be underreported.

All children with XLH should be screened for craniosynostosis at diagnosis, with specific evaluation at each clinic visit.^(^ ^19^ ^)^ Craniosynostosis should be considered in children ≤5 years of age who have XLH and an insufficient increase in head circumference, abnormal head shape, or neurological signs or symptoms.^(^ ^2^ ^)^ Alternatively, if a child develops craniosynostosis at 4 to 6 months of age, it should be considered that they may have a coexistent metabolic disorder.^(^ ^21^, ^23^ ^)^ In addition, Haffner and colleagues recommend that, after initial diagnosis of XLH, further work‐up, aimed at diagnosing the presence of rare complications caused by craniosynostosis, should be carried out for patients of all ages. In symptomatic adults and children (such as those with persistent headaches, neck pain, vomiting, or abnormal skull shape), Haffner and colleagues recommend evaluation by fundoscopy, brain MRI, or skull and/or spinal MRI to exclude craniosynostosis in children and the sequelae of craniosynostosis in adults.^(^ ^2^ ^)^

### Craniosynostosis complications in patients with XLH

The main complications associated with craniosynostosis are CM1, headaches, papilledema, syringomyelia, strabismus, learning difficulties, and seizures.^(^ ^3^, ^22^, ^41^ ^)^

CM1 is associated with craniosynostosis, although it can occur independently.^(^ ^33^ ^)^ CM1 can be asymptomatic or it can lead to the development of headaches, syringomyelia, neck pain, peripheral motor defects, peripheral sensory defects, and lower cranial nerve dysfunction.^(^ ^3^, ^33^, ^42^ ^)^

Caldemeyer and colleagues examined 16 patients with XLH and found that 7 had developed CM1, although they did not examine their patients for craniosynostosis.^(^ ^41^ ^)^ Rothenbuhler and colleagues were the only group to systematically investigate the presence of CM1 with craniosynostosis in a cohort of children with XLH. They reported craniosynostosis in 26 of 44 patients (59%), with 9 of the 26 patients with craniosynostosis (35%) having CM1. They also reported that, of the 10 patients with XLH and CM1, 9 had craniosynostosis, and there was a significant association of CM1 with fusion of the sagittal suture.^(^ ^33^ ^)^

Papilledema was reported in 12 of the case studies (Table 2 and Fig. 3*B*)^(^ ^3^, ^4^, ^5^, ^12^, ^20^, ^22^, ^24^, ^38^ ^)^ due to increased ICP caused by craniosynostosis. Papilledema can cause the blurring of vision and optic nerve atrophy, and, if left untreated, vision may be permanently affected.^(^ ^19^, ^24^ ^)^ Those with craniosynostosis should be routinely evaluated for papilledema to ensure timely detection of raised ICP.^(^ ^3^, ^4^, ^5^, ^20^, ^24^ ^)^

Headaches were reported in 8 of the XLH case studies (Table 2 and Fig. 3*B*). Headaches occurred with raised ICP, CM1, or syringomyelia and were usually present in the more advanced cases of craniosynostosis.^(^ ^3^, ^4^, ^5^, ^22^, ^31^ ^)^ Nearly all of these patients required cranioplasty surgery, which alleviated the headaches.^(^ ^3^, ^4^, ^5^, ^22^, ^31^ ^)^

Syringomyelia was reported in 3 cases in this review (Table 2 and Fig. 3*B*).^(^ ^3^, ^33^ ^)^ Syringomyelia can result in headaches, neck pain, peripheral motor defects, peripheral sensory defects, and lower cranial nerve dysfunction.^(^ ^2^, ^3^, ^33^, ^42^ ^)^ Because of the occurrence of syringomyelia together with CM1 and headaches, patients with syringomyelia often undergo surgery.^(^ ^3^, ^33^ ^)^

Strabismus can also present as a complication of craniosynostosis in patients with XLH but is less common and was present in only 3 patients identified in this review.^(^ ^5^, ^38^ ^)^

Seizures were present in only one patient in this review.^(^ ^22^ ^)^ This was a difficult‐to‐treat case and the patient required several cranial surgeries. Although it was uncertain that craniosynostosis caused the seizures, it was reported that symptoms improved postoperatively.

Learning difficulties appeared to be uncommon and arose in only one patient in this review: it was reported that one patient suffered a decline in school performance; however, this may have been complicated by his headaches and migraines.^(^ ^3^ ^)^

Many of the complications, if not all, were related to elevated ICP, which was reported in 13 of the patients with XLH, most of whom needed surgery (Table 2 and Fig. 3*B*).^(^ ^4^, ^20^, ^22^, ^24^, ^31^ ^)^ These complications caused discomfort in the patients—for example, through experiencing frequent headaches—and could cause lifelong disability through the loss of vision due to untreated papilledema.^(^ ^3^, ^4^ ^)^ Early treatment of craniosynostosis should prevent the development of these complications.^(^ ^19^ ^)^ This indicates the importance of investigating the presence of craniosynostosis and elevated ICP in patients with XLH to reduce patient suffering and improve quality of life (QoL).

### Impact of craniosynostosis on patient burden

There were no reports in the literature on the direct effect of craniosynostosis on physical function and QoL in patients with XLH; however, it is clear that, in the absence of XLH, craniosynostosis has a large impact on the health‐related QoL of affected children and their parents, both physically and psychosocially.^(^ ^43^, ^44^ ^)^ A systematic review by Park and colleagues studied the effect of primary craniosynostosis on health‐related QoL in non‐XLH patients and found that it could negatively impact psychological well‐being, social relationships, education, employment, and physical health and functioning, but this varied depending on the severity and cause of the craniosynostosis.^(^ ^45^ ^)^ Therefore, the impact of craniosynostosis in the population of patients with XLH is an important area for future research.

### Management of craniosynostosis and its complications in patients with XLH

The treatment of craniosynostosis in those with XLH is the same as for other causes of craniosynostosis: cranial vault reconstruction. However, there are some additional considerations. Severe deformity, such as significant frontal bossing, is typically present in patients with XLH and, therefore, requires more extensive reconstruction than in typical cases of craniosynostosis without the presence of XLH.^(^ ^4^ ^)^ Moreover, patients with XLH are usually diagnosed at a later stage of development,^(^ ^4^ ^)^ when the cranial bone is thicker and more difficult to contour; there are some reports in this literature review of cranial bone thickening in patients with XLH and craniosynostosis or CM1.^(^ ^2^, ^16^, ^17^, ^25^, ^36^, ^37^ ^)^ The presence of endocortical scalloping, otherwise known as copper beaten skull, can increase the risk of dural tearing during bone removal.^(^ ^4^, ^46^ ^)^

Not all patients with XLH presenting with craniosynostosis require cranial vault reconstruction because the phenotype can be mild and asymptomatic.^(^ ^31^, ^33^ ^)^ Surgery is recommended when there is the presence of elevated ICP,^(^ ^19^, ^22^, ^24^, ^38^ ^)^ and it has been estimated that <10% require surgery,^(^ ^33^ ^)^ but this varies between clinical centers. For example, Lee and Lee described surgery on a patient with craniosynostosis at the age of 12 months, before the development of complications, whereas Rothenbuhler and colleagues reported that only 4 patients of 44 underwent surgery, all 4 of whom had developed complications.^(^ ^21^, ^33^ ^)^

There were no reports on the use of helmets to manage craniosynostosis in patients with XLH.^(^ ^3^, ^4^, ^5^, ^18^, ^20^, ^21^, ^22^, ^24^, ^31^, ^33^, ^38^ ^)^ Furthermore, the effects of pharmacological treatments for XLH on craniosynostosis have not yet been investigated and warrant further research. It is uncertain whether conventional XLH therapy with phosphate and active vitamin D analogues reduces the incidence or severity of craniosynostosis.^(^ ^4^, ^5^, ^15^, ^20^, ^24^, ^25^ ^)^ There is little information on the development of craniosynostosis in patients treated with burosumab due to the relatively recent availability of this treatment for patients. In the randomized, active‐controlled, open‐label, phase 3 trial of burosumab versus continuation of conventional therapy in children with XLH (NCT02915705), there was one serious treatment‐emergent adverse event of craniosynostosis in each treatment group.^(^ ^47^ ^)^

There are many unanswered questions on the management and treatment of craniosynostosis in patients with XLH, such as: (i) Is bone healing delayed and can it complicate surgical treatment? (ii) Does optimizing metabolic control before surgery improve surgical outcome? (iii) What medical therapy results in optimal postoperative recovery? Should conventional therapy be maintained? Should burosumab be maintained?

## Discussion

The aim of this review is to increase clinician understanding and awareness of the prevalence, presentation, diagnosis, complications, and treatment of craniosynostosis in XLH. The later age of onset and heterogenous clinical severity of craniosynostosis in XLH makes diagnosis difficult and highlights the importance of maintaining a high index of suspicion and undertaking regular clinical and possibly radiological screening even after infancy. Although the majority of children with XLH who develop craniosynostosis will do so in the first few years of life, there are reports of babies with XLH born with scaphocephaly^(^ ^4^, ^21^, ^23^ ^)^ and others with radiological evidence of craniosynostosis who remain asymptomatic and have a normal appearance for life.^(^ ^18^, ^31^, ^33^ ^)^ The late onset of complications secondary to raised ICP (CM1 and papilledema) highlights the importance of diagnosis and monitoring of craniosynostosis in all children with XLH.^(^ ^3^, ^5^, ^18^ ^)^

Haffner and colleagues recommend a detailed clinical evaluation, including evidence of rickets, growth failure, dental abnormalities, and signs of craniosynostosis and/or intracranial hypertension, as part of the initial diagnostic work‐up for XLH. Craniosynostosis should be considered in children ≤5 years of age who have XLH and an insufficient increase in head circumference, abnormal head shape, or neurological signs (including headache and vomiting as a result of increased ICP) and historical craniosynostosis should be considered in symptomatic adults (eg, patients with neurological signs, such as persistent headaches). The authors recommend evaluation by brain and/or spinal MRI.^(^ ^2^ ^)^

The complications associated with craniosynostosis can be severe if left untreated. Raised ICP leading to CM1 and syringomyelia can cause headaches, neck pain, peripheral motor defects, peripheral sensory defects, and lower cranial nerve dysfunction,^(^ ^2^, ^3^, ^33^, ^42^ ^)^ and papilledema can cause irreversible damage to vision.^(^ ^48^ ^)^

There remains much to understand about craniosynostosis in XLH. Areas for further research include understanding the true incidence of craniosynostosis in XLH, differentiating the role of hypophosphatemia and FGF23 on development of craniosynostosis, exploring the effect of medical therapy on the development and natural history of craniosynostosis in XLH, and evaluating the impact of craniosynostosis on patient QoL, psychological well‐being, and physical and cognitive development.

There have been no investigations into the effect of treatment for XLH on the development of craniosynostosis. Although children on phosphate and active vitamin D analogues may develop craniosynostosis,^(^ ^4^, ^5^, ^15^, ^20^, ^24^, ^25^ ^)^ it is uncertain if conventional medical therapy influences the prevalence or natural history. Unlike conventional therapy, burosumab treatment aims to normalize serum phosphate.^(^ ^49^ ^)^ Evaluating the real‐world effect of burosumab on craniosynostosis development and natural history will help our understanding of the impact the normalization of serum phosphate has on this complication. It is likely that the age at which burosumab is started will influence its effect on craniosynostosis. For example, at present, burosumab is not indicated for children <1 year of age in Europe or Australia,^(^ ^49^, ^50^ ^)^ whereas it is indicated for use in children ≥6 months of age in the US^(^ ^51^ ^)^ and Canada.^(^ ^52^ ^)^ It may be necessary to initiate burosumab treatment within the first 12 months of life to impact the development of craniosynostosis. The differing indications for use in countries where approval has been granted may be informative in determining the impact of the age of burosumab initiation on craniosynostosis development. The independent impact of *PHEX* mutation on craniosynostosis will be best answered in animal studies.

There have been no studies into the psychological burden of craniosynostosis nor its effects on the QoL of children and adults with XLH. However, similar to other patient groups without XLH,^(^ ^45^ ^)^ it could be expected that untreated craniosynostosis would contribute to reduced QoL in XLH.^(^ ^45^ ^)^ Reduced QoL in craniosynostosis patients without XLH has been associated with both the complications of raised ICP and the skull deformity itself.^(^ ^45^ ^)^ Addressing this gap in our understanding of craniosynostosis in the XLH community will be important to ensure that a holistic management plan is achieved.

In summary, craniosynostosis in XLH is a potentially underrecognized condition. All children with XLH should have clinical assessment for craniosynostosis ≤5 years of age, after which time development is unlikely.^(^ ^2^ ^)^ There remain important questions to be answered regarding the etiology of craniosynostosis in XLH, the impact of medical therapy on natural development and natural history, and the effect of craniosynostosis on QoL, psychological burden, and development. There are also still improvements to be made in awareness and diagnosis of craniosynostosis in patients with XLH.

## Conflicts of Interest

SW was an employee of Kyowa Kirin International at the time of writing. AW is an employee of Kyowa Kirin International. CFM has received consultant fees, speaker fees, a travel grant, and a research grant from Kyowa Kirin. AB has received consultant fees and a research grant from Kyowa Kirin.

## Author Contributions

**Craig F Munns:** Methodology; validation; writing—review and editing. **Edward P Maguire:** Formal analysis; investigation; writing—original draft; writing—review and editing. **Angela Williams:** Conceptualization; funding acquisition; methodology; project administration; supervision; writing—review and editing. **Sue Wood:** Conceptualization; funding acquisition; methodology; project administration; supervision; writing—review and editing. **Andrew Biggin:** Methodology; validation; writing—review and editing.

### Peer Review

The peer review history for this article is available at https://publons.com/publon/10.1002/jbm4.10728.
